# Metal chelating and anti-radical activity of *Salvia officinalis* in the ameliorative effects against uranium toxicity

**DOI:** 10.1038/s41598-022-20115-9

**Published:** 2022-09-23

**Authors:** Deniz Aydin, Emine Yalçin, Kültiğin Çavuşoğlu

**Affiliations:** 1 Department of Biology, Institute of Science, Giresun University, Giresun, Turkey; 2 Department of Biology, Faculty of Science and Art, Giresun University, 28200 Giresun, Turkey

**Keywords:** Cell biology, Physiology, Plant sciences

## Abstract

Uranium is a highly radioactive heavy metal that is toxic to living things. In this study, physiological, cytogenetic, biochemical and anatomical toxicity caused by uranium and the protective role of sage (*Salvia officinalis* L.) leaf extract against this toxicity were investigated with the help of *Allium* test. Germination percentage, root length, weight gain, mitotic index (MI), micronucleus (MN) formation, chromosomal aberrations (CAs), superoxide dismutase (SOD) and catalase (CAT) enzyme activities, malondialdehyde (MDA) levels and changes in root meristem cells were used as indicators of toxicity. In the experimental stage, a total of six groups, one of which was the control, were formed. Group I was treated with tap water, while group II and III were treated only with sage (190 mg/L and 380 mg/L). Groups IV, V and VI were germinated with uranyl acetate dihydrate (0.1 mg/mL), uranyl acetate dihydrate + 190 mg/L sage and uranyl acetate dihydrate + 380 mg/L sage, respectively. *Allium cepa* L. bulbs of each group were germinated for 72 h, and at the end of the period, routine preparation techniques were applied and physiological, cytogenetic, biochemical and anatomical analyzes were performed. As a result, uranium application caused a significant decrease (p < 0.05) in all physiological parameters and MI values. MN, CAs numbers, SOD and CAT enzyme activities and MDA levels increased significantly (p < 0.05) with uranium application. Uranium promoted CAs in the root tip cells in the form of fragment, vagrant chromosome, sticky chromosome, bridge and unequal distribution of chromatin. In addition, it caused anatomical damages such as epidermis cell damage, cortex cell damage and flattened cell nucleus in root tip meristem cells. Sage application together with uranium caused significant (p < 0.05) increases in physiological parameters and MI values and significant decreases in MN, CAs, SOD and CAT activities and MDA levels. In addition, the application of sage resulted in improvement in the severity of anatomical damages induced by uranium. It was determined that the protective role of sage observed for all parameters investigated was even more pronounced at dose of 380 mg/L. The protective role of sage against uranium toxicity is related to its antioxidant activity, and sage has 82.8% metal chelating and 72.9% DPPH removal activity. As a result, uranyl acetate exhibited versatile toxicity in *A. cepa*, caused cytotoxicity by decreasing the MI rate, and genotoxicity by increasing the frequencies of MN and CAs. And also, Sage acted as a toxicity-reducing agent by displaying a dose-dependent protective role against the toxic effects induced by uranyl acetate.

## Introduction

Heavy metal is a general term for a group of metals and metalloids with an atomic density of more than 4 g/cm^3^ or more than 5 times that of water. Heavy metals that are serious environmental pollutants and have toxic effects on organisms include lead (Pb), cadmium (Cd), aluminum (Al), zinc (Zn), mercury (Hg), arsenic (As), silver (Ag), chromium (Cr), copper (Cu), iron (Fe) and uranium (U)^1^. The enhanced use of heavy metals in various industries such as mining, refinery, nuclear power plants, plastic, textile and paper processing, agricultural sector, pharmaceutical, domestic and technological applications increases the amount of heavy metal emitted into the environment. It is also known that natural events such as weather conditions and volcanic eruptions contribute significantly to heavy metal pollution^2^. All these processes cause environmental pollution by metals in air, water and soil, and the contamination of heavy metals to organisms accelerates. In recent years, there has been an increasing ecological and global public health concern due to environmental pollution caused by these metals. Heavy metals that enter different organisms through respiration, nutrition, contact and diffusion cause toxic effects by accumulating in various tissues. In biological systems, heavy metals damage cellular organelles such as mitochondria, lysosomes, endoplasmic reticulum, nucleus, and cell membranes. It also affects some enzymes involved in detoxification and damage repair, interacts with DNA and nuclear proteins, causing conformational changes that can lead to DNA damage, cell cycle modulation, carcinogenesis and apoptosis^3,4^. Heavy metals also have toxic effects on plants due to their high reactivity and directly affect growth, germination and energy production processes, especially in plants. Reduction in plant germination and growth is the most important and primary manifestation of metal toxicity in plants. Heavy metals also cause toxic effects on plant physiology and metabolism. This toxicity has a wide spectrum of action, from inhibition of enzymatic activity to mutagenesis^5^. Heavy metals, which increase the production of reactive oxygen species (ROS), cause leaf yellowing, stunting, yield reduction, chlorophyll deterioration and lipid peroxidation in plants, and prevent the uptake of macro elements by plants^6^. Uranium, an important heavy metal, is a radioactive heavy metal estimated to have formed in supernovas about 6.6 billion years ago. It is easily oxidized in air and is covered with an oxide layer. Therefore, in nature, uranium is mainly found in the oxidized state. It takes place in the structure of many minerals such as uraninite, carnotite, otunite, uranophan, torbernite and coffin. It is also found in lignite, monazite sands, phosphate rock and phosphate fertilizers. Due to the use of phosphate fertilizers in the agricultural sector, the amount in the soil is increasing day by day^7^. Uranium and its derivatives are used as x-ray radiation shielding in hospitals, as counterweights for the rudders and wings of commercial aircraft and forklifts, and in the keels of sailing yachts^8^. Uranium, which is naturally occurring and the heaviest element, is found in the earth's crust at an average concentration of 0.0003% (3 mg/kg). Its concentration in seawater is about 3 µg/L. Due to its presence in the air, soil, rocks, surface and underground waters, it reaches as much as plants, animals and humans^7,9^. Uranium has serious toxic effects on organisms. It causes regression of weight gain, reduction of chlorophyll content and inhibition of photosynthetic electron transfer in plants^10^. In humans, it causes pathological effects by accumulating in bones, liver, kidneys and other tissues^11^. In this study, a comprehensive toxicity profile of uranyl acetate in *Allium cepa* L., a bioindicator plant, was created by using multiple parameters and the toxicity-reducing effect of the antioxidant *Salvia officinalis* L. (sage) was investigated.

*Salvia* is the largest genus of the Lamiaceae family and has about 900 species all over the world. Some members of this genus are widely used in the manufacture of perfumes and cosmetics, mayonnaise, meat products, pie dough and various types of food, such as salad dressing. *S. officinalis* is the most well-known and researched species of the genus *Salvia*. It is a perennial, round-shaped, shrub-shaped herb and is native to the Middle East and Mediterranean regions. Compounds such as carnosic acid, carnosol, rosmarinic acid, tannin, flavonol, anthocyanin and camphor determined in its phytochemical content enable sage to exhibit strong biological and pharmacological activity^12–14^. The high radical scavenging capacity, prevention of lipid peroxidation, protection of endogenous antioxidant levels, antioxidant and anti-inflammatory effects of sage may be associated with the phytoactive compounds it contains^15,16^. The protective effects of sage against the toxicity of many chemicals have been investigated in the literature, but there is no study on its protective feature against uranium toxicity.

In this study, the toxic effects of uranium, which contaminates agricultural areas due to the use of phosphate fertilizers, on the indicator plant *A. cepa* and the protective role of sage against this toxicity were also investigated. The toxicity profile and the toxicity-reducing role of sage were investigated with multiple parameters. In *A. cepa*, physiological effects were investigated by using parameters related to germination, biochemical effects were investigated by examining malondialdehyde (MDA) level, superoxide dismutase (SOD) and catalase (CAT) levels, and cytogenetic effects were investigated using mitotic index (MI), micronucleus (MN) and chromosomal aberrations (CAs) analyses. 2,2-diphenyl-1-picrylhydrazyl (DPPH) removal effect and metal chelating activities were also investigated to support the protective property of sage.

## Materials and methods

### Test materials and experimental groups

*A. cepa* bulbs of approximately the same size, purchased from a commercial market in Giresun province, were used as test material. Uranyl acetate dihydrate (CAS number: 6159-44-0) and other chemicals were obtained from Sigma-Aldrich. Sage leaf extract (380 mg, 90 capsules) used as a protective biological product was commercially obtained from Sepe Natural Company. The content of sage consists entirely of natural extract obtained from the leaf of the plant. There is no filler used in the capsule content. Sage doses were determined by considering the daily consumption amounts recommended by nutritionists, and 380 mg/L, which is the amount of sage contained in a capsule, and 190 mg/L, which is half of it, were tested in the study. The dose of uranyl acetate dihydrate used in the study was determined on the basis of literature studies and was preferred in the dose range where uranium compounds have toxic effects on plants^17,18^. Experimental research on plants, including the procurement of plant material, complies with institutional, national and international guidelines and legislation.

From the bulbs, 6 groups were formed as indicated below, with 10 bulbs in each group.

**Table:** 

Group I	Control
Group II	190 mg/L sage
Group III	380 mg/L sage
Group IV	0.1 mg/mL uranyl acetate dihydrate
Group V	0.1 mg/mL uranyl acetate dihydrate + 190 mg/L sage
Group VI	0.1 mg/mL uranyl acetate dihydrate + 380 mg/L sage

**Figure 1:**
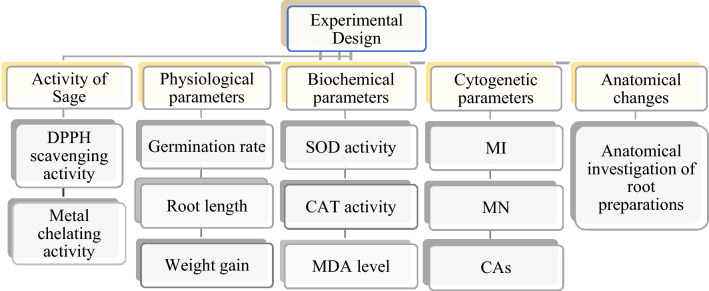
Experimental stages of study. *DPPH* 2,2-diphenyl-1-picrylhydrazyl, *SOD* superoxide dismutase, *CAT* catalase, *MDA* malondialdehyde, *MI* mitotic index, *MN* micronucleus, *CAs* chromosomal aberrations.

Bulbs were placed in sterile glass beakers with a diameter of 85 × 100 mL. Those in the control group were germinated with tap water, and those in the treatment group with 0.1 mg/mL dose of uranyl acetate dihydrate and 190 mg/L and 380 mg/L doses of sage for 72 h at room temperature. During the germination period, daily controls of the beakers were made and solution additions were made when necessary. At the end of the germination period, the bulbs were washed with distilled water and made ready for toxicity tests^19^. The parameters tested during the study are summarized in Fig. 1.

### DPPH scavenging and metal chelating activity of sage

Powders contained in commercially available sage capsules were extracted before experimental procedures. 10 g of sage powder was incubated with 200 mL of dH_2_O for 12 h at room temperature. The residues obtained after evaporation of the solvent at the end of the incubation were used as extracts in all experimental stages. DPPH removal effect and metal chelating activities of extract were investigated to support the protective property of sage.

DPPH scavenging activity of sage was determined using the method suggested by Gündüz et al.^20^ 2.4 mL DPPH (0.1 mM) solution in methanol was mixed with 1.6 mL sage. The absorbance of the solution left in the dark for 30 min was measured spectrophotometrically at 517 nm. Butylated hydroxyanisole (BHA) and butylated hydroxytoluene (BHT) were used as standards and DPPH radical scavenging activity was calculated using Eq. (1).

1 $$\% {\text{ DPPH radical scavenging activity }} = \, \left( {{\text{A}}_{{\text{c}}} - {\text{ A}}_{{\text{s}}} } \right)/{\text{A}}_{0} \times { 1}00$$

A_c_ is the absorbance of the control and A_s_ is the absorbance of the sage or standard solution. The experiment was repeated three times at each concentration.

The metal chelating activity of sage was determined with the protocol suggested by Dinis et al.^21^. 50 μL of FeCl_2_ was mixed with sage (25, 50, 100, 200 mg/mL). Then 5 mM ferrozine (0.2 mL) was transferred to the mixture to start the reaction. The mixture was shaken vigorously and stored at room temperature for approximately 10 min. The absorbances of mixtures were measured at 562 nm.

Metal chelating activity of sage (%) was calculated using Eq. (2)

2 $$\% {\text{ Chelating activity }} = \, [{1 } - \, \left( {{\text{A}}_{{\text{s}}} /{\text{A}}_{{\text{c}}} } \right) \, \times { 1}00$$

A_c_ is the absorbance of the control and A_s_ is the absorbance of the sage or standard solution. The experiment was repeated three times at each concentration.

### Physiological parameter measurements

The effects of uranium and sage applications on root growth in *A. cepa* were investigated according to radicle formation with the help of a millimetric ruler, and the effects on live weight were investigated by measuring the bulb weights with the help of precision balances before and after the application. The effects on bulb germination were calculated using Eq. (3)^22^.

3 $${\text{Germination}}\,\,{\text{percentage}}\,\,(\% ) = {\text{Number}}\,\,{\text{of}}\,\,{\text{germinated}}\,\,{\text{seeds}}/{\text{Total}}\,\,{\text{number}}\,\,{\text{of}}\,\,{\text{seeds}} \times 100$$

### Genotoxicity tests

Root tips in each group were cut about 1 cm long to determine CAs and MN formation. It was kept in “Clarke” solution prepared from 3 volumes of ethyl alcohol (C_2_H_5_OH) and 1 volume of glacial acetic acid (CH_3_COOH) for 2 h. Afterward, it was washed in C_2_H_5_OH (96%) for 15 min and stored at + 4 °C in C_2_H_5_OH (70%). Root tips were hydrolyzed in 1 N hydrochloric acid (HCl) for 17 min in an oven at 60 °C, and kept in CH_3_COOH (45%) for 30 min. In the last stage, root tips were stained with aceto-carmine for 24 h, crushed in CH_3_COOH (45%) and examined under the IRMECO IM-450 TI model research microscope, and CAs and MN formations were photographed at × 500 magnification^23^.

MN evaluation Fenech et al.^24^ according to the proposed criteria:

- MN size should be 1/3 of the cell nucleus,
- MN should be round or oval shaped,
- MN cell should be distinguishable from the nucleus. In case of contact between the MN and the nuclear membranes, the boundary between them should be clearly defined.

Mitotic index (MI), expressed as the percentage of cells undergoing mitosis, was calculated using Eq. (4).

4 $${\text{MI }} = {\text{ Number of cells undergoing mitosis }}{/}{\text{ Total number of cells }} \times { 1}00$$

MN and CAs were calculated by analyzing 1000 cells in each group, and MI by analyzing 10,000 cells in each group.

### Biochemical analysis

#### MDA measurements

MDA levels were measured according to the method suggested by Unyayar et al.^25^. In summary, 1 mL of 5% trichloroacetic acid (C_2_HCl_3_O_2_) solution was added to 0.5 g of fresh root tip and homogenized. The homogenate was transferred to a new sterile tube and centrifuged for 10 min at 12,000*g* at 24 °C. Equal volumes of 5% thiobarbituric acid (C_4_H_4_N_2_O_2_S) and supernatant were transferred to a new sterile tube and incubated in 20% C_2_HCl_3_O_2_ solution at 96 °C for 30 min. It was then placed in an ice bath and centrifuged at 10,000*g* for 5 min. The absorbance of the supernatant was measured at 532 nm and the MDA level was shown as μM/g FW.

#### Enzyme activity measurements

Enzyme extraction was carried out at + 4 °C. 0.5 g of fresh root tips were washed with distilled water, homogenized in 5 mL of mono sodium phosphate (NaH_2_PO_4_) buffer (50 mM, pH 7.8) and centrifuged at 10,500*g* for 20 min. In the last step, the supernatant was stored at + 4 °C until analysis^26^.

#### SOD activity measurement

SOD activities were measured using the method proposed by Beauchamp and Fridovich^27^. Briefly, the reaction solution consisting of 1.5 mL 0.05 M NaH_2_PO_4_ buffer, 0.3 mL 130 mM methionine (C_5_H_11_NO_2_S), 0.3 mL 750 μM nitroblue tetrazolium chloride (C_4_OH_3_OCl_2_N_10_O6), 0.3 mL 0.1 mM EDTA (C_10_H_16_N_2_O_8_)-Na_2_, 0.3 mL 20 μM riboflavin (C_17_H_2_ON_4_O_6_), 0.01 mL enzyme extract, 0.01 mL 4% insoluble polyvinylpyrrolidone (C6H9NO)n and 0.28 mL of de-ionized water was prepared. The reaction process was started by placing the tubes under two pieces of 15 W fluorescent lamps for 10 min and ended by keeping the tubes in the dark for 10 min. The absorbance was read at 560 nm and the SOD activity was shown as U/mg FW^26^.

#### CAT activity measurement

CAT activities were measured using the method proposed by Beers and Sizer^28^. In summary, CAT activity was measured using UV–VIS spectrophotometer at 24 °C in 2.8 mL of reaction solution consisting of 0.3 mL of 0.1 M hydrogen peroxide (H_2_O_2_), 1.0 mL of distilled water and 1.5 mL of 200 mM NaH_2_PO_4_ buffer. The reaction process was initiated with the addition of 0.2 mL enzyme extract. CAT activity was measured by following the decrease in absorbance at 240 nm as a result of H_2_O_2_ consumption and was shown as OD_240nm_ min/g^26^.

#### Determination of root meristematic cell damages

The root tips, which were cut about 1 cm in length, were washed with distilled water, placed between the foam material, and their cross sections were taken in one move with the help of a pre-sterilized, sharp razor blade. The sections were stained with 5% methylene blue on a slide for 2 min, closed with the help of entellen, and turned into a permanent preparation. The slides were examined under the IRMECO IM-450 TI model research microscope; meristematic cell damage was detected and photographed at × 200 magnification^29^.

### Statistical analysis

Statistical evaluation of the data was carried out using the SPSS Statistics 22 (IBM SPSS, Turkiye) package program. Data are shown as mean ± standard deviation (SD). The statistical significance between the means was determined with the help of One-way ANOVA (one-way analysis of variance) and Duncan tests, and it was considered statistically significant when the determined value (p) was less than 0.05.

## Result and discussion

### DPPH scavenging and metal chelating activity of sage

**Figure 2:**
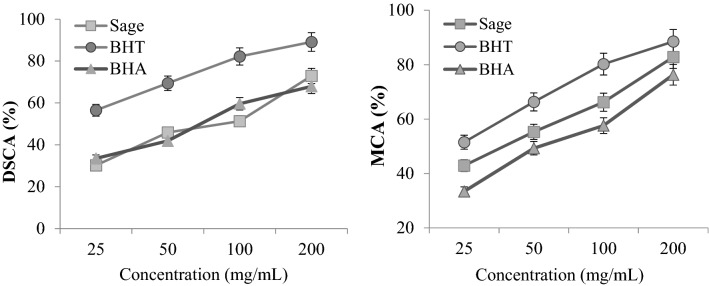
DPPH scavenging (DSCA) and metal chelating activity (MCA) of sage.

In order to support the protective role of sage against uranyl acetate toxicity, DPPH scavenging and metal chelating activities, which indicate antioxidant activity, were investigated and the results are given in Fig. 2. DPPH is a stable free radical in aqueous solutions, and the decrease in the absorbance of the DPPH radical indicates antioxidant activity. Sage was determined to exhibit a dose-dependent increasing DPPH scavenging effect. DPPH scavenging activities of 200 mg/mL sage, BHA and BHT were determined as 72.9%, 67.9% and 89.1%, respectively. The metal chelating activities of sage and standards were determined by evaluating their ability to compete with ferrozine for the ferrous ions. A dose-dependent increasing activity was also obtained in metal chelating activity. Metal chelating activities of 200 mg/mL sage, BHA and BHT were determined as 82.8%, 76.3% and 88.5%, respectively. These results show that sage has a free radical scavenging activity that is higher than the standard antioxidant BHA and lower than BHT. Its high DPPH removal and metal chelating activity indicate the antiradical and metal chelating properties of sage, as well as its strong antioxidant capacity. There are also studies in the literature that draw attention to the similar features of sage. Emre et al.^30^ reported that different *Salvia* species grown in Turkiye exhibited metal chelating activity in the range of 45.0–80.48%. Roman et al.^31^ investigated the antiradical properties of *S. officinalis* extract and stated that it exhibited more than 85% DPPH removal activity. With the powerful antioxidant property, Sage has a protective role against much toxicity, and the results obtained in the *Allium* test in this study confirm this hypothesis.

### Physiological parameters

The effects of uranyl acetate and sage application on selected physiological parameters are shown in Table 1. The maximum germination percentage, root length and weight gain were measured in the control group and Group II and Group III, which were exposed to two different doses of sage. No statistically significant difference was found between the physiological parameter values measured in these groups (p > 0.05). In Group IV, in which 0.1 mg/mL of uranyl acetate was administered, statistically significant decreases were found in all investigated physiological parameter values compared to the control group (p < 0.05). It was observed that this decrease was approximately 2.1 times for germination percentage, about 7 times for root length and about 4.8 times for weight gain. The application of sage together with uranyl acetate caused a statistically significant (p < 0.05) increase in the values of all investigated physiological parameters, although not as much as the control group. It was determined that these increases were more pronounced at the 380 mg/L dose of sage. Compared to Group IV, germination percentage increased approximately 1.2 times, root length approximately 1.9 times and weight gain approximately 1.8 times in Group VI.

**Table 1:** Effects of uranyl acetate and sage application on selected physiological parameters.

Groups	Germination (%)	Root length (cm)	Initial weight (g)	Final weight (g)	Weight gain (g)
Group I	100	6.30 ± 2.82^a^	7.41 ± 1.85	13.75 ± 2.92	+ 6.34^a^
Group II	100	6.20 ± 2.80^a^	7.49 ± 1.88	13.90 ± 2.94	+ 6.41^a^
Group III	100	6.40 ± 2.90^a^	7.35 ± 1.81	13.80 ± 2.90	+ 6.45^a^
Group IV	46	0.90 ± 1.13^d^	7.45 ± 1.86	8.78 ± 1.95	+ 1.33^d^
Group V	53	1.70 ± 1.24^c^	7.50 ± 1.93	9.92 ± 1.97	+ 2.42^c^
Group VI	62	2.90 ± 1.38^b^	7.43 ± 1.87	11.38 ± 2.11	+ 3.95^b^

Group I: Control, Group II: 190 mg/L sage, Group III: 380 mg/L sage, Group IV: 0.1 mg/mL uranyl acetate, Group V: 0.1 mg/mL uranyl acetate + 190 mg/L sage, Group VI: 0.1 mg/mL uranyl acetate + 380 mg/L sage. Data are shown as mean ± SD. 50 bulbs were used to determine germination percentage, and 10 bulbs were used to determine root length and weight gain. The averages shown with different letters^(a–d)^ in the same column are significant at p < 0.05.

Although there is no comprehensive study in the literature on the effects of uranium or uranyl acetate application on the physiological properties of plants, there are some studies on the effects of other heavy metals. For example, Çavuşoğlu et al.^32^ determined that Pb and Hg heavy metal application at 10 and 50 ppm doses caused dose-dependent decreases in the germination percentage, root length and weight gain of *Cicer arietinum* L. seeds. They also reported that these decreases were more pronounced in the group exposed to the 50 ppm dose of Hg. Çavuşoğlu and Yalçın^33^ determined that 25 and 50 ppm doses of Al and Co application caused a dose-dependent decrease in the germination percentage, root length and weight of *Phaseolus vulgaris* L. cv. kidney bean seeds. They also observed that these decreases were more pronounced at the 50 ppm dose of Al. Gürel et al.^34^ observed that 2.4, 8.0 and 12.5 mg/L Cr doses caused dose-related decreases in germination percentage, root length and weight gain in *A. cepa*. Girasun et al.^35^ determined that Pb application at 50, 100 and 200 mg/L doses caused a dose-dependent decrease in physiological parameters such as germination percentage, root length and weight gain in *A. cepa*. Macar et al.^36^ found statistically significant reductions in germination percentage, root length and weight gain in *A. cepa* bulbs exposed to 5.5 mg dose of Co for 72 h.

In this study, it is thought that the abnormalities in physiological parameters as a result of uranium exposure are due to the reduction of *A. cepa* roots' intake of water and inorganic substances. Because it has been reported in the literature that high doses of heavy metal exposure in different plant species reduce the water and mineral substance uptake of the roots, and their productivity decreases by affecting the photosynthesis reactions and nitrogen metabolisms. On the other hand, it has been reported that exposure to heavy metals causes root, shoot, plant growth and plant weight reduction, deterioration of grana structure, inhibition of chlorophyll synthesis and respiration and development of apoptosis and necrosis processes in plants. ROS produced by heavy metals is shown as the main reason for the processes that encourage all these negative effects in the plant. Sage, which exhibits strong DPPH removal and metal chelating activity, protected against oxidative stress induced by uranium and exhibited a toxicity-reducing effect with its antioxidant property. It has also been stated in the literature that plants have developed some effective defense mechanisms to combat ROS-induced oxidative stress^37^. Therefore, it is considered that these defense mechanisms developed by *A. cepa* to prevent uranium from entering the cell may be another reason for the decrease in the investigated physiological parameter values. Because the excessive increases in the number/frequency of epidermis and cortex cells observed in the microscopic examination of root tip meristematic cells support this idea.

### Genotoxicity parameters

**Figure 3:**
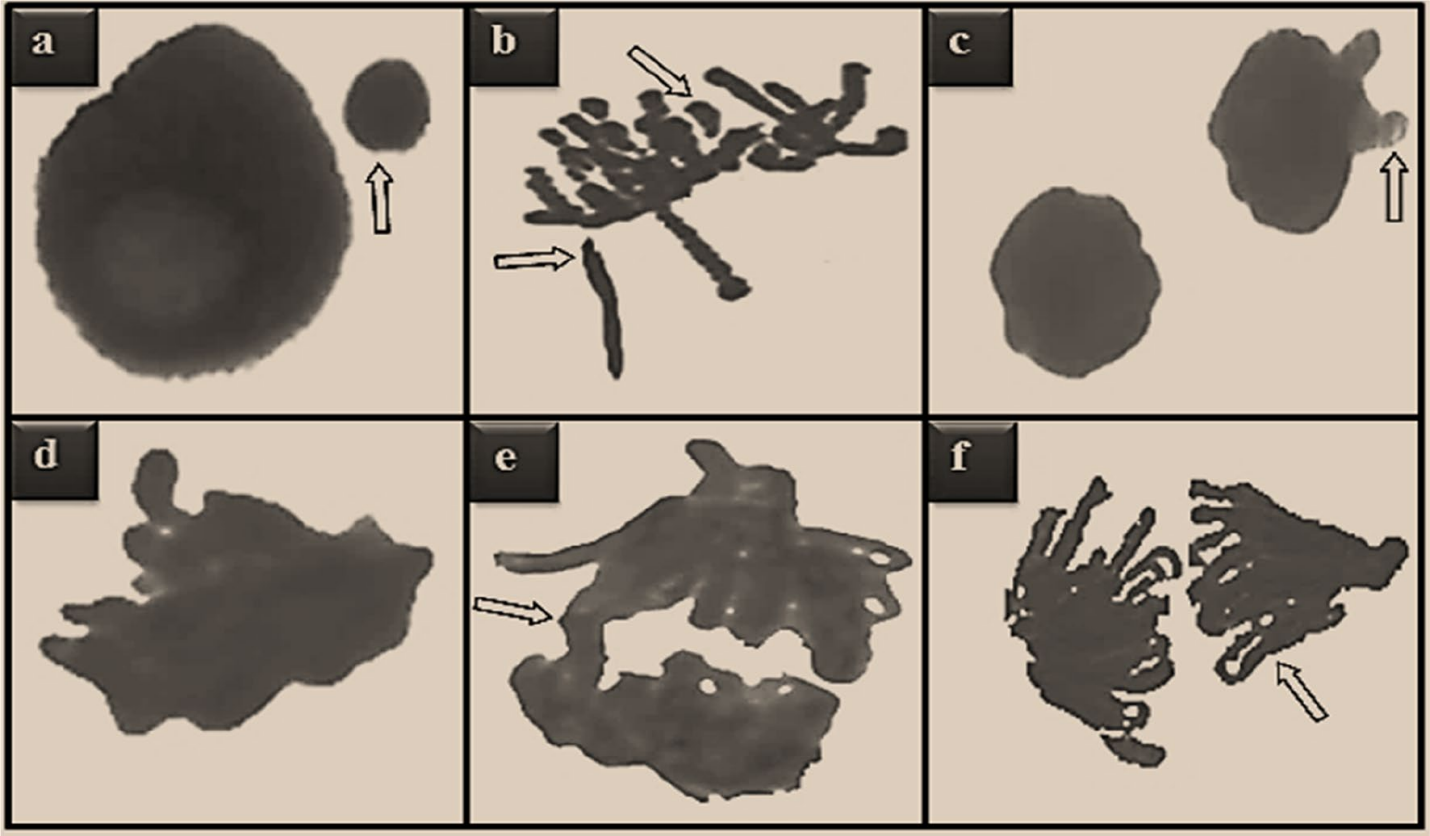
CAs induced by uranyl acetate. MN in interphase (**a**), fragment in metaphase (**b**), vagrant chromosome in anaphase (**c**), sticky chromosome in prophase (**d**), bridge in early anaphase (**e**), unequal distribution of chromatin in anaphase (**f**).

**Figure 4:**
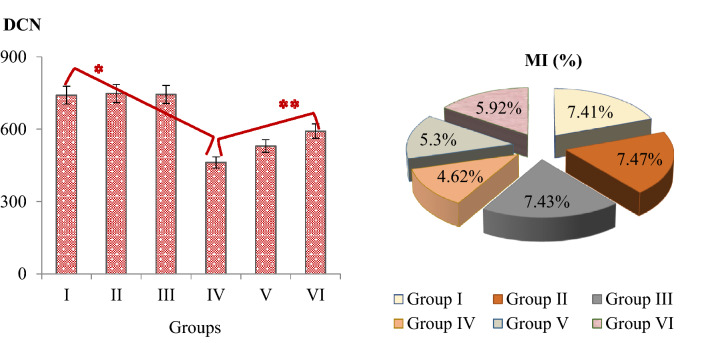
The effects of uranyl acetate and sage on DCN and MI (%). Group I: Control, Group II: 190 mg/L sage, Group III: 380 mg/L sage, Group IV: 0.1 mg/mL uranyl acetate, Group V: 0.1 mg/mL uranyl acetate + 190 mg/L sage, Group VI: 0.1 mg/mL uranyl acetate + 380 mg/L sage. MI was calculated by counting 10,000 cells in each group. *indicates statistical difference between Groups I and IV, **indicates statistical difference between Groups IV and VI (*p* < 0.05). DCN: dividing cell number, MI: mitotic index.

The genotoxicity induced by uranyl acetate application and the protective role of sage against this toxicity are shown in Figs. 3, 4 and Table 2. Statistically insignificant (p > 0.05) MN formations were found in the control group and Group II and Group III, which were exposed to two different doses of sage. In addition, CAs in the form of a few sticky chromosome and unequal distribution of chromatin was detected in these groups, which was not statistically significant (p > 0.05). On the other hand, the highest MI value (741.30, 747.90 and 743.80, respectively) was also determined in these groups. The application of 0.1 mg/mL uranyl acetate caused the highest rate (82.40) of MN formation (p < 0.05) in the root tip cells of the bulbs in Group IV, and promoted CAs such as fragment, vagrant chromosome, sticky chromosome, bridge and unequal distribution of chromatin and caused significant decreases (p < 0.05) in the MI value. The greatest effect of uranyl acetate application on chromosomes occurred in the form of fragment formation. The application of sage together with uranyl acetate decreased the genotoxic effects of uranyl acetate, and caused a statistically significant (p < 0.05) decrease in the frequencies of MN and CAs, and a significant (p < 0.05) increase in the MI value, depending on the dose. It was determined that these alterations observed in the investigated genotoxic parameters were more pronounced in Group VI, where 380 mg/L dose of sage was administered. Compared to Group IV, the frequency of fragment decreased approximately 1.5 times, the MN frequency decreased approximately 1.4 times, and the MI rate increased approximately 1.3 times in Group VI.

**Table 2:** Protective role of sage against uranyl acetate-induced genotoxicity.

Damages	Group I	Group II	Group III	Group IV	Group V	Group VI
MN	0.32 ± 0.46^d^	0.28 ± 0.35^d^	0.21 ± 0.30^d^	82.40 ± 6.13^a^	68.20 ± 5.32^b^	56.90 ± 4.75^c^
FRG	0.00 ± 0.00^d^	0.00 ± 0.00^d^	0.00 ± 0.00^d^	63.20 ± 5.16^a^	50.60 ± 4.84^b^	41.50 ± 4.26^c^
VC	0.00 ± 0.00^d^	0.00 ± 0.00^d^	0.00 ± 0.00^d^	49.70 ± 3.98^a^	40.10 ± 3.38^b^	32.60 ± 2.91^c^
SC	0.42 ± 0.64^d^	0.36 ± 0.48^d^	0.32 ± 0.44^d^	38.50 ± 3.21^a^	29.40 ± 2.70^b^	21.60 ± 2.38^c^
B	0.00 ± 0.00^d^	0.00 ± 0.00^d^	0.00 ± 0.00^d^	26.30 ± 1.94^a^	20.10 ± 1.60^b^	14.20 ± 1.18^c^
UDC	0.16 ± 0.28^d^	0.12 ± 0.26^d^	0.00 ± 0.00^d^	20.40 ± 1.66^a^	14.80 ± 1.27^b^	6.30 ± 1.92^c^

Group I: Control, Group II: 190 mg/L sage, Group III: 380 mg/L sage, Group IV: 0.1 mg/mL uranyl acetate, Group V: 0.1 mg/mL uranyl acetate + 190 mg/L sage, Group VI: 0.1 mg/mL uranyl acetate + 380 mg/L sage. Data are shown as mean ± SD (n = 10). MN and CAs were calculated by counting 1000 cells in each group. The averages shown with different letters^(a–d)^ in the same line are important at p < 0.05.

*MN* micronucleus, *FRG* fragment, *VC* vagrant chromosome, *SC* sticky chromosome, *B* bridge, *UDC* unequal distribution of chromatin.

Although there is no comprehensive study in the literature on genotoxicity caused by exposure to uranium or uranyl acetate in plants, there are some studies conducted with experimental animals. For example, Çavuşoğlu et al.^38^ observed MN formation in erythrocyte and buccal mucosal epithelial cells of Swiss albino mice exposed to 5 mg/kg b.w of uranyl acetate by oral gavage for 5 days. In addition, they reported a decrease in MI value with CAs in the form of break, fragment, gap, acentric and ring chromosomes in bone marrow cells. In addition, there are some studies in the literature investigating the genotoxicity induced by other heavy metal ions in plants. For example, Çavuşoğlu et al.^32^ determined that exposure to Pb and Hg at two different doses (10 and 50 ppm) caused an increase in the frequency of MN in *C. arietinum* root tip cells and promoted CAs in the form of sticky chromosome and bridge. Çavuşoğlu and Yalçın^33^ observed that exposure to Al and Co at 25 and 50 ppm doses caused MN formation in *P. vulgaris* cv. kidney bean root cells. Gürel et al.^34^ reported that administration of three different doses of Cr (2.4, 8.0 ve 12.5 mg/L) caused a dose-dependent decrease in MI in *A. cepa* root tip cells. In addition, they found an increase in the frequency of MN and the numbers of CAs such as fragments, unequal distribution of chromatin, sticky chromosomes, bridges, reverse polarization and c-mitosis. Girasun et al.^35^ showed that exposure to three different doses (50, 100 ve 200 mg/L) of Pb decreased the MI value, increased the frequency of MN, and caused damage in the form of fragments, adhesions, bridges and c-mitosis in *A. cepa* root tip cells, depending on the application dose. Macar et al.^36^ observed a decrease in MI, an increase in MN formation and an increase in the number of CAs in root tip cells of *A. cepa*, where 5.5 mg of Co was applied. They also determined that Co application promotes CAs in the form of fragment, sticky chromosome, bridge, unequal distribution of chromatin, multipolar anaphase, nucleus damage, and irregular mitosis.

In our study, it is thought that the main reason for the decrease in MI value and the increase in the numbers of MN and CAs in Group IV treated with uranyl acetate may be due to the direct or indirect interaction of uranium with chromosomes. Because it has been reported in the literature that heavy metals disrupts the structure of DNA directly or indirectly by producing ROS, promoting DNA damages. On the other hand, some heavy metals have been reported to cause disruptions in DNA repair processes. For example, while Cr causes damage by reacting directly with DNA, As, Ni and Cd act by preventing the repair processes of DNA double-strand breaks. Damages such as MN, fragments, breaks, sister chromatid exchanges and variation are other CAs promoted by heavy metal ions^39^. The genotoxic and cytotoxic effects induced by uranyl acetate may be related to the occurrence of oxidative stress in general. Sage protects the integrity of the genome by reducing the oxidative load in the cell, especially with its strong metal chelating activity and antioxidant power. The reductions in MN and CAs frequencies observed in groups V and VI treated with sage + uranyl acetate confirm this idea.

### Biochemical parameters

**Figure 5:**
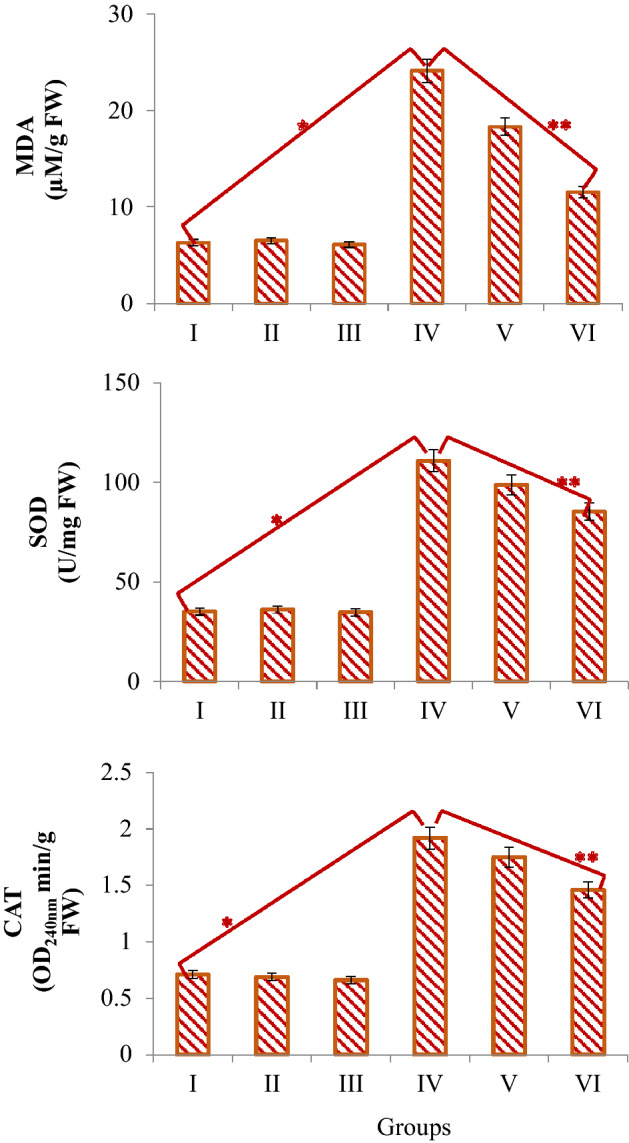
Effect of uranyl acetate and sage application on selected biochemical parameters. Group I: Control, Group II: 190 mg/L sage, Group III: 380 mg/L sage, Group IV: 0.1 mg/mL uranyl acetate, Group V: 0.1 mg/mL uranyl acetate + 190 mg/L sage, Group VI: 0.1 mg/mL uranyl acetate + 380 mg/L sage. * indicates statistical difference between Groups I and IV, ** indicates statistical difference between Groups IV and VI (*p* < 0.05).

The effects of uranyl acetate and sage application on selected biochemical parameters are shown in Fig. 5. No statistically significant difference was observed between the root MDA levels and SOD and CAT activities of the control group and Group II and Group III exposed to two different doses of sage (p > 0.05). Uranyl acetate application at 0.1 mg/mL dose caused statistically significant (p < 0.05) increases in root MDA level, which is an indicator of lipid peroxidation, and in SOD and CAT activities, which are antioxidant enzymes. Compared to the control group, these increases were found to be approximately 3.8 times for MDA level, approximately 3.2 times for SOD activity and approximately 2.7 times for CAT activity in Group IV. It was determined that the application of sage together with uranyl acetate again promoted statistically significant (p < 0.05) decreases in MDA levels, SOD and CAT activities, depending on the dose. These decreases were even more pronounced in Group VI exposed to 380 mg/L of sage. Compared to Group IV, approximately 2.1-fold decrease in MDA level, approximately 1.3-fold decrease in SOD activity and approximately 1.3-fold decrease in CAT activity was detected in Group VI.

Although there is no comprehensive study in the literature of biochemical toxicity induced by exposure to uranium or uranyl acetate in plants, there are some studies with Swiss albino mice. Çavuşoğlu et al.^38^ reported a significant increase in blood MDA levels and a significant decrease in GSH levels in Swiss albino mice exposed to 5 mg/kg b.w of uranyl acetate by oral gavage for 5 days. In a similar study, Yapar et al.^40^ found significant increases in MDA levels and significant decreases in GSH levels in liver and kidney tissues of Swiss albino mice exposed to 5 mg/kg b.w of uranyl acetate. In addition, there are some studies dealing with the biochemical toxicity induced by other heavy metals other than uranium in plants. Çavuşoğlu et al.^32^ reported that MDA levels in *C. arietinum* root tip cells exposed to Pb and Hg heavy metals at 10 and 50 ppm doses increased dose-dependently, and these increases were even more pronounced at 50 ppm doses of Hg. Çavuşoğlu and Yalçın^33^ stated that the application of Al and Co at two different doses (25 and 50 ppm) caused dose-related increases in the MDA levels of *P. vulgaris* cv. kidney bean root cells, and these increases were higher et al. doses than at Co doses. Macar et al.^36^ observed that Co application at 5.5 mg dose caused significant increases in MDA levels and SOD and CAT enzyme activities of *A. cepa* root tip cells.

MDA is a 3-carbon aldehyde, which is one of the most important markers of cell membrane damage, in other words, lipid peroxidation. Lipid peroxidation is a reaction caused by free radicals that cause oxidative damage of unsaturated fats. A free radical can then abstract the H atom and form an oxidized lipid free radical, producing a peroxyl radical. The peroxyl radical can remove an electron and produce a lipid hydroperoxide and another lipid free radical. This process can continue as a chain reaction. Since lipid hydroperoxide is unstable, it decomposes to form MDA and 4-hydroxy-2-nonenal products. In cases where the increase of free radicals in the cell, enzyme systems and antioxidant molecules in the cell are not sufficient for protection, these free radicals attack cell membranes, cause lipid peroxidation and increase MDA levels^41^. Therefore, the increase in MDA levels in *A. cepa* root cells treated with uranyl acetate can be explained by the fact that uranium causes free radical production and these free radicals cause damage to the membranes of the root cells.

SOD and CAT enzymes are known as antioxidants that prevent the formation of free radicals in the cell or eliminate or neutralize their effects. While the SOD enzyme neutralizes the superoxide radical, the CAT enzyme catalyzes the conversion of H_2_O_2_, which is highly toxic for the cell, into water and oxygen^42^. Therefore, the increase in SOD and CAT enzyme levels in *A. cepa* root cells exposed to uranyl acetate can be explained by the fact that uranium causes free radical production and increases SOD and CAT enzyme levels as a defense mechanism of the cell to minimize the harmful effects of free radicals. The fact that uranyl acetate increases MDA levels and induces antioxidant enzyme activities can be explained by triggering oxidative stress. The decrease observed in MDA, SOD and CAT levels in Group V and Group VI treated with sage + uranyl acetate shows that sage provides protection against the biochemical toxicity of uranyl acetate. This healing property is closely related to the antioxidant, antiradical and metal chelating activities of sage.

### Anatomical observations

**Figure 6:**
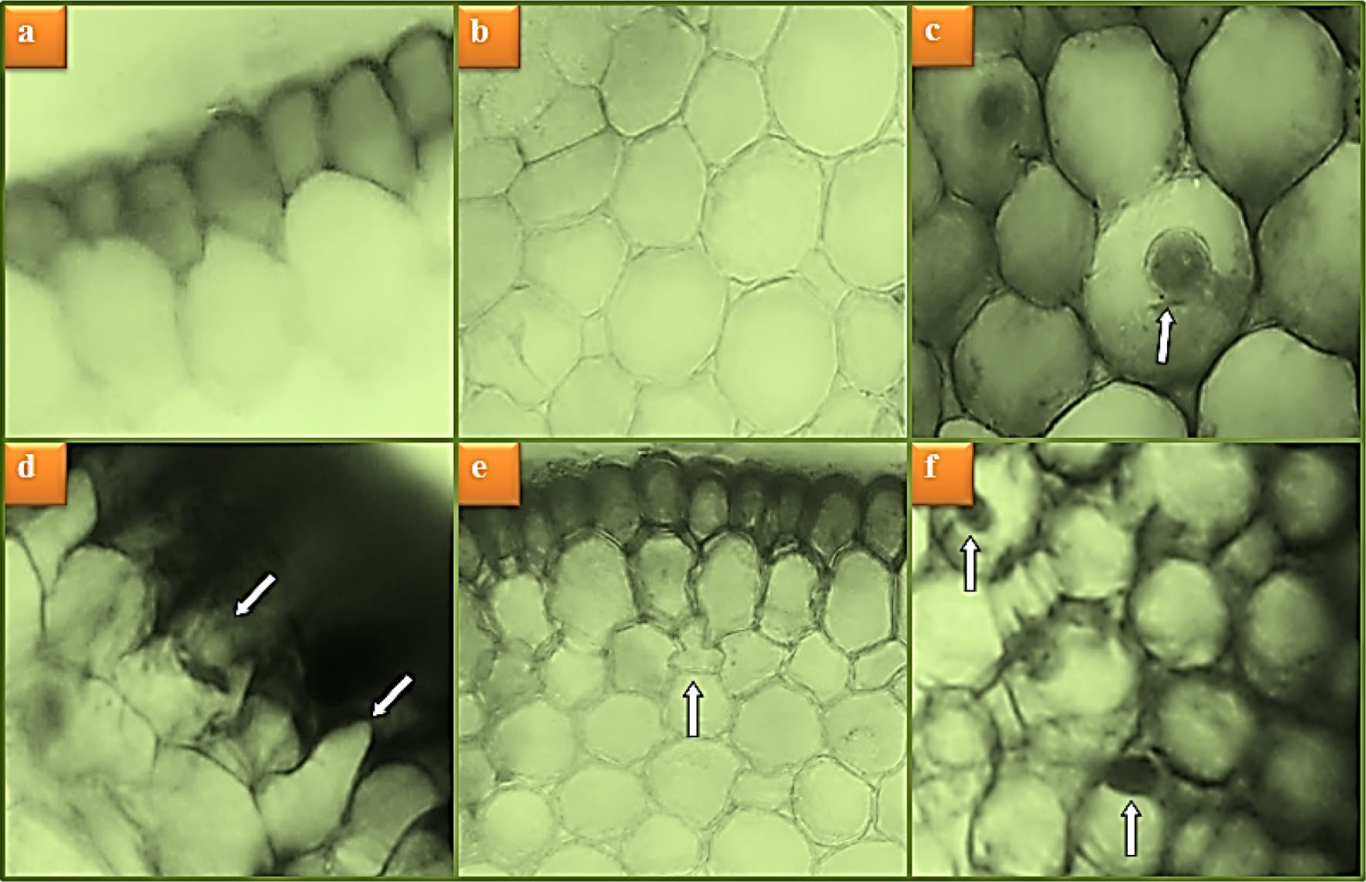
Meristematic cell damages induced by uranyl acetate. Normal appearance of epidermis cells (**a**), normal appearance of cortex cells (**b**), normal appearance of cell nucleus-*oval* (**c**), epidermis cell damage (**d**), cortex cell damage (**e**), flattened cell nucleus (**f**).

The effects of uranyl acetate and sage application on the root anatomy of *A. cepa* are shown in Fig. 6 and Table 3. No damage was observed in the root meristem cells of Group II and Group III, which were exposed to two different doses of sage with the control group. In Group IV exposed to uranyl acetate at a dose of 0.1 mg/mL, epidermis and cortex cell damage, as well as meristematic cell damage in the form of flattened cell nucleus were observed. Co-administration of sage with uranyl acetate caused reductions/improvements in the severity of observed meristematic cell damage by reducing the negative effects of uranyl acetate, depending on the dose. It was determined that this decrease in the severity level was more pronounced at 380 mg/L dose of sage.

**Table 3:** Protective role of sage against meristematic cell damages induced by uranyl acetate.

Groups	ECD	CCD	FCN
Group I	**−**	**−**	**−**
Group II	**−**	**−**	**−**
Group III	**−**	**−**	**−**
Group IV	**+++**	**++**	**+++**
Group V	**++**	**+**	**++**
Group VI	**+**	**−**	**+**

*Group I: Control, Group II: 190 mg/L sage, Group III: 380 mg/L sage, Group IV: 0.1 mg/mL uranyl acetate, Group V: 0.1 mg/mL uranyl acetate + 190 mg/L sage, Group VI: 0.1 mg/mL uranyl acetate + 380 mg/L sage. ECD: epidermis cell damage, CCD: cortex cell damage, FCN: flattened cell nucleus. (−): no damage, (+): little damage, (++): moderate damage, (+++): severe damage.

Although there is no study in the literature that deals with the anatomical changes caused by uranium or uranyl acetate exposure in plant root tip meristematic cells, there are some studies on the anatomical effects of other heavy metals. Gürel et al.^34^ reported that 2.4, 8.0 and 12.5 mg/L Cr doses caused anatomical damage in the form of cell deformation, thickening of the cortex cell wall, flattened cell nucleus and necrosis in root tip meristematic cells of *A. cepa*. They also stated that the severity of these damages was dose dependent. Çavuşoğlu et al.^43^ observed anatomical damage such as cell deformation, necrosis, flattening cell nucleus, thickening of the cortex cell wall, inclearly vascular tissue and accumulation of some substances in cortex cells in *A. cepa* root tip meristematic cells exposed to Hg at 25, 50 and 100 mg/L doses. Girasun et al.^35^ detected cell damage such as thickening of the cortex cell wall, cell deformation, inclearly vascular tissue and necrosis in *A. cepa* root tip meristem cells of 50, 100 and 200 mg/L doses of Pb exposure, the severity of which increased with the application dose. Macar et al.^36^ reported that 5.5 mg Co dose promoted damages such as epidermis cell deformation, thickening of the cortex cell wall and flattened cell nucleus in *A. cepa* root tip meristematic cells.

This suggests that this epidermis and cortex cell damage induced by uranium occurs as a result of the defense mechanisms developed by plants against heavy metal ions. Because the roots have increased the number and frequency of the epidermis and cortex cells in order to prevent uranium from entering the cell, and these damages may have occurred as a result of the compression/suppression of the cells. The information in the literature that plants develop different defense mechanisms against heavy metal toxicity, such as accumulation, storage and crystallization of metals in certain regions, or changes in the cell membrane and cell wall, increase in vacuole numbers and metal-binding protein synthesis^44^, supports our this idea.

### Protective role of sage

In recent studies, different plant extracts such as lycopene, carotene, *Ginkgo biloba* L., green coffee, green tea and stinging nettle are used to reduce toxicity promoted by toxic agents such as heavy metal ions. In this study, sage treatment provided significant protection against the physiological, biochemical, cytogenetic and anatomical abnormalities exhibited by the application of uranyl acetate in the *A. cepa* root tip cells. It provided improvement in germination-related parameters such as root length and weight gain, and decreased MN and CAs frequencies, which were detected at high rates after uranyl acetate application. These improvements increased depending on the dose and the highest protection was obtained at the dose of 380 mg/L. In this study, it was determined that sage has antiradical property and scavenges the DPPH radical at a rate of 72.9%. Sage is a powerful antioxidant compound, which also exhibits an important metal chelating activity. These powerful properties of sage are related to the active ingredients it contains. The greatest role in the protective role of sage is the antioxidant activity exhibited due to phenolic compounds such as carnosic acid, carnosol, rosmarinic acid and camphor in the content. There are some studies in the literature focused on the antioxidant role of sage. For example, Lima et al.^45^ investigated the antioxidant potential of traditional water infusion (tea) of sage in vivo in mice and rats. In conclusion, it was determined that replacing the water in the diet of rodents with sage for 14 days did not affect the body weight and food consumption of the animals. They also reported that sage did not cause liver toxicity, liver GST activity was increased in rats (24% rate) and mice (10% rate) drinking sage, on the other hand, sage caused an improvement in the antioxidant status of hepatocytes, increased GSH levels and provided a protection against lipid peroxidation. Horváthová et al.^46^ investigated the protective effect of sage extract against oxidative stress to which liver cells of Sprague–Dawley rats are exposed. As a result, no negative effects were observed on basal DNA damage levels and SOD activities in hepatocyte cells of animals that drank sage for 14 days, and no changes were detected in the biochemical parameters of blood plasma. On the contrary, they determined that sage extract significantly increased GPx activity, decreased DNA damage levels caused by oxidants, and provided antioxidant protection by increasing GSH levels. Alshubaily and Jambi^47^ investigated the possible protective role and antioxidant activity of sage extract against metabolic disorders caused by hypercholesterolemic diet in heart and testicular tissues of rats. In conclusion, they determined that the hypercholesterolemic diet significantly increased serum lipid content, cardiac marker enzyme activities, MDA levels, and significantly decreased high-density lipoprotein-cholesterol levels in testes and heart tissues. They observed that the co-administration of hypercholesterolemic diet and sage extract reduced the damage caused by the hypercholesterolemic diet by causing a decrease in lipid peroxidation, induction of heart and testis functions, and increased activity. They reported that essential oil, phenolic contents and other antioxidant components contained in sage extract were effective in this.

## Conclusion

Increasing industrialization and agricultural practices make the frequent use of chemicals and their contamination to the environment inevitable. As a result of the frequent use of phosphate fertilizers in the agricultural sector, uranium, the amount of which increases in the soil, also contaminates plant products and the living things that consume these products. In this study, the toxic effects of uranium on the indicator plant *A. cepa* and the protective role of sage against this toxicity were investigated. In conclusion, administration of 0.1 mg/mL uranyl acetate caused toxicity by promoting statistically significant changes in physiological, cytogenetic, biochemical and anatomical parameters in *A. cepa*. Uranyl acetate showed a cytotoxic effect by causing a decrease in MI rates, and a genotoxic effect by increasing the frequency of MNs and CAs. Co-administration of sage with uranyl acetate at doses of 190 mg/L and 380 mg/L reversed the toxic effects of uranyl acetate and resulted in an improvement in the values of all the parameters studied. It was determined that these improvements were even more pronounced at 380 mg/L sage dose. It was determined that 200 mg/mL sage showed 72.9% DPPH removal activity and 82.8% metal chelating activity, and these results indicate the strong antioxidant activity. The protective role of sage is related to the antioxidant activity that occurs as a result of the cumulative effect of the active ingredients it contains. This study will lead to many similar studies and will draw attention to the fact that every chemical compound polluting the environment adversely affects organisms and that many natural extracts can be a solution against this toxicity.

## Data Availability

The datasets used and/or analyzed during the current study are available from the corresponding author on reasonable request.

## Abbreviations

BHA: Butylated hydroxyanisole
BHT: Butylated hydroxytoluene
DCN: Dividing cell number
DPPH: 2,2-Diphenyl-1-picrylhydrazyl
CAs: Chromosomal aberrations
MDA: Malondialdehyde
MI: Mitotic index
MN: Micronucleus
SOD: Superoxide dismutase
CAT: Catalase
ROS: Reactive oxygen species
